# Eco-friendly nanoparticles from *Fusarium solani* suppress biofilms and quorum sensing in *Pseudomonas aeruginosa*†

**DOI:** 10.1039/d5na00898k

**Published:** 2025-11-10

**Authors:** Karokh Ali Khdir, Sirwan Muhsin Muhammed

**Affiliations:** a Biology Department, College of Education, University of Sulaimani Sulaymaniyah Kurdistan Region 46001 Iraq karokh.khdir@univsul.edu.iq; b Biology Department, College of Science, University of Sulaimani Sulaymaniyah Kurdistan Region 46001 Iraq sirwan.muhammed@univsul.edu.iq

## Abstract

The rise in antibiotic resistance among biofilm-producing *Pseudomonas aeruginosa* has renewed interest in alternative strategies to fight drug resistance by synthesizing effective and affordable nanoparticles (NPs). In this context, the study aims to assess the antibacterial, antibiofilm, and anti-quorum-sensing properties of biogenic AgNPs and CuNPs against *P. aeruginosa*. NPs were synthesized using *Fusarium solani* and characterized through various spectroscopic techniques. *P. aeruginosa* was identified using the BD Phoenix system and the 16S rRNA gene. The antibacterial and antibiofilm assays were performed using 96-well microtiter plates, and QRT-PCR was adopted to assess the impact of the NPs on quorum-sensing genes. The data of UV–vis spectroscopy clarified the surface plasmon resonance nature of AgNPs at 415–420 nm and CuNPs at 280 nm. SEM and TEM confirmed spherical NPs with average sizes of 17 nm for AgNPs and 21 nm for CuNPs. Additionally, XRD indicated a face-centered cubic structure with crystallite sizes from 18 to 26 nm, and EDS analysis revealed that silver and copper are the major constituents in the nanostructures, with weight percentages of 78.2% and 53.1%, respectively. FTIR revealed the contribution of various functional groups. The MIC ranged from 31.25 to 125 μg mL^−1^, and the MBC ranged from 125 to 250 μg mL^−1^. The maximum biofilm inhibition ranged from 53.3 to 84.23%, and the maximum biofilm disruption of preformed biofilm ranged from 51.4 to 76.13%. AgNPs downregulated LasI, LasR, RhlI, RhlR, PqsABCDE, and PqsR genes by 1.4–13-fold, and the CuNPs downregulated these genes by 1.3–11-fold, but PqsR was upregulated by 0.91–2-fold. *F. solani* mediated AgNPs and CuNPs demonstrated the multi-target action of these NPs and suggest promising avenues for their application as antibacterial and antibiofilm agents against drug-resistant pathogens.

## Abstract 2

Biogenic AgNPs and CuNPs from *F. solani* demonstrated potent antibiofilm and anti-quorum-sensing against XDR *P. aeruginosa*, highlighting their therapeutic potential.

## Graphical abstract

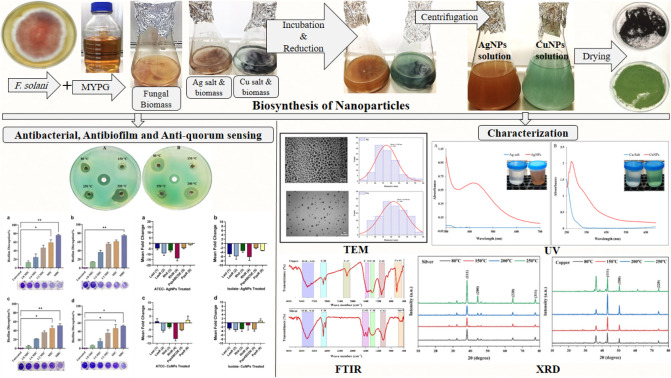

## 1 Introduction

Bacterial infections are among the major contributors to global health concerns, resulting in increased persistence of these infections, treatment failures, and higher morbidity and mortality.^1,2^ The overcoming of many infections around the world has become a significant problem owing to the widespread development of drug resistance in microorganisms.^3^ Nowadays, antimicrobial resistance (AMR) has become a significant public health problem worldwide. Approximately 1.27 million people died from AMR in 2019, and almost 5 million died from drug-resistant bacterial infections in 2022.^4^*Pseudomonas aeruginosa* is one of the most common pathogens implicated in drug-resistant infections. This Gram-negative, aerobic, motile bacillus belongs to the *Pseudomonadaceae* family. *P. aeruginosa* can thrive in both abiotic and biotic environments. It can be isolated from nosocomial infections, cystic fibrosis (CF), wounds, burns, pulmonary and urinary tract infections (UTIs), as well as health-care devices such as dialysis equipment, respirators, inhalers, and vaporizers.^5^*P. aeruginosa* is a multidrug-resistant (MDR) infectious agent that causes approximately 80% of opportunistic infections and poses a serious threat to patients suffering from cancer, burns, and cystic fibrosis, which causes death in nearly half of all cases.^6^

Concerning drug resistance, the intrinsic, acquired, and adaptive are the main resistance mechanisms in *P. aeruginosa* strains.^7^ The adaptive mechanism involves the establishment of biofilm as a multicellular bacterial community stabilized by an extracellular polymeric substance (EPS) and adhered to different abiotic and biotic surfaces.^8^ The potential mechanisms of antibiotic resistance in biofilms include delayed or improper penetration of antibiotics into the biofilms due to the presence of EPS, slow growth of bacteria in biofilms, and alteration of the microenvironment inside the biofilms, and the bacteria may differentiate toward a protective phenotype, which lowers the sensitivity of biofilms.^9^ The fundamental mechanism that regulates the biofilm formation is quorum sensing (QS). Four quorum-sensing systems have been recognized in *P. aeruginosa*: the lasI/lasR, which utilizes autoinducer 3-oxododecanoyl-l-homoserine lactone, the rhlI/rhlR, which uses *N*-butanoyl homoserine lactone, PqsABCDE/PqsR, which uses 2-heptyl-3-hydroxy-4-quinolone, and the last one is AmbBCDE/IqsR, which utilizes 2-(2-hydroxyphenyl) thiazole-4-carbaldehyde.^10,11^ Furthermore, the QS in *P. aeruginosa* regulates other virulence factors like enzymes, toxins, motility, pili, and pigments. These factors are major contributors to pathogenicity and antimicrobial resistance.^12^ Due to previous resistance mechanisms, many strains of *P. aeruginosa* have gained resistance to various antibiotics, including piperacillin/tazobactam, ceftazidime, carbapenems, aminoglycosides, fluoroquinolones, and polymyxins.^13^

It has been proven that most conventional antibiotics fail to treat drug-resistant pathogens and are unable to penetrate and disrupt biofilms.^14^ Therefore, the intervention of non-antibiotic therapeutics as an alternative approach to combat drug-resistant bacterial infections and the discovery of novel antibiofilm agents are crucial goals in current research. Presently, one of the promising and innovative approaches is nanotechnology. Various nanoparticles (NPs), including silver, copper, zinc and, gold, have been synthesized and applied in biomedical science. They received considerable attention, with an emphasis on numerous life-threatening diseases.^15^ Copper nanoparticles (CuNPs) and silver nanoparticles (AgNPs) have demonstrated antibacterial potential toward drug-resistant Gram-negative and positive bacteria because of their nanoscale sizes, higher ratio of surface area to volume, and unique physicochemical properties.^16,17^ AgNPs have a broad array of applications in cosmetic products, the food industry, and composite fibers.^18^ The biomedical applications of AgNPs include antibacterial and antibiofilm activities.^19^ AgNPs are also utilized in dental care and medical devices to avoid the growth of bacteria.^20^ CuNPs are highly reactive and can interact with other particles owing to their higher surface area, which increases their broad range of antimicrobial efficacy.^21^

In fact, various biological and physicochemical techniques are recommended for the fabrication of NPs.^22^ The biogenic method employed by fungi and other organisms has numerous advantages over physicochemical methods as it is affordable, environmentally friendly, and can be processed at ordinary temperature and pressure, while exhibiting higher bioactivity and lower toxicity. Also, metabolites secreted by biological entities act as reducing mediators that reduce the metal salts to corresponding NPs and act as capping agents that give stability and bio-compatibility to the NPs for different biomedical applications.^23^ Additionally, the biosynthesis of NPs by fungi of the genus *Fusarium* is more preferable due to the easier acquisition of sufficient biomass, higher heavy metal tolerance, and bulk extracellular secretion, and the NP synthesis is sustainable and extracellular, thereby lowering the downstream cost. Among the fungal species in the genus *Fusarium*, *F. oxysporum* and *F. solani* have been identified as potential candidates.^24^

In this study, biogenic CuNPs and AgNPs were obtained by the reduction of copper nitrate and silver nitrate using *F. solani* as a bio-reducing agent and then characterized. Furthermore, the antibacterial, antibiofilm, and anti-quorum-sensing effectiveness of the NPs were evaluated against a standard strain and a clinical isolate of *P. aeruginosa*. To our knowledge, this is the first report demonstrating the dual activity of *F. solani*-mediated CuNPs and AgNPs in effectively targeting biofilms and quorum sensing in the extensively drug-resistant (XDR) strain of *P. aeruginosa*.

## 2 Materials and methods

### 2.1 Materials

Copper nitrate (CuNO_3_) was purchased from Biochem Chemopharma (France), and silver nitrate (AgNO_3_) was purchased from CARLO ERBA Reagents (Italy). Ethanol and Milli-Q water (resistivity of 18.5 MΩ) were purchased from Merck (Germany) and used to wash the nanoparticles. Flat-bottom cell culture plates from Sorfa Life Science, China, and crystal violet from Biochem Chemopharma, France were used. Culture media was purchased from Merck and Sigma-Aldrich (Germany), and HiMedia (India). The glassware was cleaned with Milli-Q water.

### 2.2 Methods

#### 2.2.1. Conventional and molecular identification of *P. aeruginosa*

*P. aeruginosa* ATCC 9027 and a clinical isolate were used. The clinical isolate was isolated from a patient with deep wound infection from Hospital of Smart Health Tower (Madam Mitterrand Street, Sulaymaniyah, Iraq), and identified using conventional bacteriological techniques as well as the automatic BD Phoenix™ M50 identification system (USA). Additionally, the 16S rRNA was employed for further identification. *P. aeruginosa* ATCC and the isolate were grown on nutrient agar for 24 hours. DNA was extracted from fresh colonies using the AddPrep Bacterial Genomic DNA Extraction Kit (Addbio, Korea) following the manufacturer's protocol. The 16S rRNA gene was amplified using primers PA-SS-F (5′-GGGGGATCTTCGGACCTCA-3′) and PA-SS-R (5′-TCCTTAGAGTGCCCACCCG-3′).^25^ Amplification of the target gene was accomplished in a reaction mixture composed of 10 μl of Add-Taq master mix (Addbio, Korea), 3 μl of DNA samples, 10 pmol of each primer, and 5 μl of high-performance liquid chromatography-grade H_2_O. PCR cycle conditions were controlled as follows: denaturation for 5 min at 95 °C, followed by 40 cycles of amplification, each consisting of denaturation at 94 °C for 20 seconds, annealing at 60 °C for 30 seconds, and extension at 72 °C for 20 seconds. A final extension was performed for 5 min at 72 °C. The products of the PCR reaction were electrophoresed, ethidium bromide-stained, and visualized using a gel documentation system (Biobase, China).^25,26^

#### 2.2.2. Synthesis of AgNPs and CuNPs

*Fusarium solani* was previously isolated from the Tanjaro/Kurdistan region of Iraq.^27^ This local isolate was employed in the biosynthesis of AgNPs and CuNPs. *F. solani* was inoculated on potato dextrose agar. After sufficient growth, the fresh colonies were added to Erlenmeyer flasks containing sterilized malt-yeast-peptone-glucose rich medium (MYPG), which consisted of malt extract 3 g, yeast extract 3 g, peptone 2 g, and glucose 10 g L^−1^. The flasks were incubated at 28 °C for 6 days in a shaker incubator (150 rpm). Aseptically, the fresh mycelia of *F. solani* were collected using a refrigerated centrifuge (Sorvall Biofuge Stratos, Thermo, Germany) at 5000 rpm for 10 min at 4 °C, and washed several times. The biomass was weighed and utilized for the biosynthesis of NPs. Five Erlenmeyer flasks were prepared; the first flask contained 100 mL of 10 mM AgNO_3_, and the second flask contained 100 mL of 10 mM CuNO_3_ (the salt solutions were filtered through a 0.22 μm millipore syringe filter). These flasks were supplied with 10 g of washed biomass. The third flask contained 100 mL of 10 mM AgNO_3_, and the fourth flask contained 100 mL of 10 mM CuNO_3_ without biomass (these were the negative controls). The fifth flask contained 10 g of biomass and 100 mL of distilled water (it served as the second negative control). All flasks were incubated at 28 °C in the dark for 6 days in a shaker incubator (150 rpm). Following incubation, the solutions were filtered and centrifuged at 10 000 rpm for 10 min. After that, the supernatants were decanted into sterile containers, dried, and calcined at 80 °C, 150 °C, 200 °C, and 250 °C. Finally, the calcined NPs were washed three times with 70% ethanol, dried, and then subjected to characterization.^28,29^

## 3 Characterization of nanoparticles

### 3.1 UV-visible spectroscopy

The bioreduction of silver and copper salts into respective AgNPs and CuNPs was monitored using a UV-vis spectrophotometer EMC-11-UV (EMCLAB, Germany) in the range of 200–800 nm. In brief, biogenic NPs were suspended in sterile distilled water and then added to a quartz cuvette to investigate the surface plasmon resonance of the NPs.^30^

### 3.2 Fourier transform infrared (FTIR) spectroscopy

The infrared absorption and emission spectra for the predictable functional groups involved in NP biosynthesis were measured using FTIR. Biogenic NPs were freeze-dried and suspended with potassium bromide (KBr, ≥ 99.0%), mixed thoroughly, and then examined in the range of 400–4000 cm^−1^ with FTIR (Nicolet iS10 FTIR Spectrometer, USA).^16^

### 3.3 X-ray diffraction (XRD)

The crystalline nature of NPs was observed using an X-ray diffractometer (Philips X'pert Pro MPD, Netherlands) with Ni-filtered Cu Kα radiation (*λ* = 1.54 Å). The X-ray scanning was carried out over a range of theta values from 0° to 80° at 40 kV voltage and 30 mA current.^31^

### 3.4 Transmission electron microscopy (TEM), scanning electron microscopy (SEM), and energy-dispersive spectroscopy (EDS)

The morphological properties of biogenic NPs, comprising shape, size, and distribution, were evaluated using TEM (ZEISS, Germany). The powder of NPs was dispersed in ultra-pure water (MiliQ water) through ultrasonication, and a few drops of the suspension were loaded on a copper grid coated in carbon. The TEM images were obtained at 80 kV voltage. SEM (ZEISS SIGMA VP/Germany) was used to examine the topography and surface properties of the NPs. Energy-dispersive spectroscopy (X-Max Oxford, England) was employed to conduct elemental analysis and identify qualitative and quantitative chemical compositions of biogenic NPs.^32^

## 4 Antibacterial activity of nanoparticles

### 4.1 Agar well diffusion

The well diffusion was employed to assess the preliminary antibacterial potential of biogenic CuNPs and AgNPs against *P. aeruginosa* using Moller-Hinton agar (MHA). The fresh culture was used to prepare an adjusted bacterial suspension of 0.5 McFarland (1–2 × 10^8^ CFU mL^−1^) using a DensiCHEK Plus (bioMérieux, France). A volume of 100 μl from the bacterial suspension was spread uniformly throughout the medium with a cotton swab. After that, wells with a 6 mm diameter were made using a glass Pasteur pipette. A volume of 100 μl of each NP solution annealed at different annealing temperatures was loaded onto the wells (NP solutions were ultrasonicated before use). The standard antibiotic colistin 10 μg disc^−1^ is a positive control. Finally, the plates were incubated for 24 hours at 37 °C. The day after, inhibitory zone diameters were measured and tabulated.^33^

### 4.2 Broth microdilution

A broth microdilution was employed to evaluate minimum inhibitory (MIC) and minimum bactericidal (MBC) concentrations of AgNPs and CuNPs using 96-well microtiter plates. Aseptically, the treated wells of 96-well plates were prepared by loading the wells with 190 μL of Mueller-Hinton broth (MHB) containing different concentrations of NPs and 10 μL of adjusted bacterial suspension. The final concentrations of NPs ranged from 1.95 to 500 μg mL^−1^. Untreated wells contained MHB and bacterial suspension without NPs. The wells were gently shaken to mix the contents, and then the plates were incubated at 37 °C for 24 hours with continuous shaking (150 rpm) using a shaker incubator (Grant-bio, UK). The MIC was estimated visually and then spectrophotometrically using a Microplate reader (Bio-TEK, USA) at 600 nm and calculated by comparing the optical density (OD) of treated wells with that of untreated ones. The MIC is the minimum concentration of the NPs at which the bacteria do not demonstrate any observable growth. To estimate the MBC, 5 μL was taken from the wells, inoculated on nutrient agar, and incubated for 24 hours at 37 °C till growth was seen in the untreated subculture. The MBC is defined as the minimum concentration of the NPs at which the tested bacteria were completely killed, with growth inhibition >99.9%.^34,35^ The antibacterial activity of NPs was assessed in triplicate and repeated across three independent experiments. The growth inhibition percentages were calculated as follows:
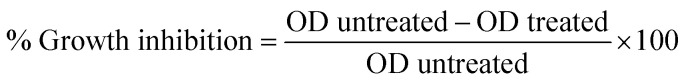


## 5 Biofilm formation assessment

At first, the ability of *P. aeruginosa* strains to produce biofilms was evaluated in a 96-well flat-bottom microtiter plate using the same method as mentioned later in the biofilm inhibition assay. The degree of biofilm formation was determined using the following equations: (1) biofilm formation = OD of CV (inoculated wells) − OD of CV (negative control wells).^36^ (2) OD value of a tested strain = average OD of a strain − cutoff value of negative control (ODc).^37^

## 6 Antibiofilm activity of nanoparticles

### 6.1 Biofilm inhibition assay

This assay was conducted to determine the effect of sub-MIC concentrations of AgNPs and CuNPs on the inhibition of *P. aeruginosa* biofilm formation using crystal violet (CV) as described previously.^38^ Briefly, the wells of 96-well microtiter plates were loaded with Moller-Hinton broth (190 μl) and 10 μl of a bacterial suspension and then treated with 1/2, 1/4, and 1/8 MIC of NPs. Untreated wells contained MHB and standard bacterial suspension without NPs. Following incubation at 37 °C for 24 hours, the individual well contents were carefully discarded and rinsed three times by normal saline to remove non-adherent cells. The biofilm produced by adherent bacteria was fixed with 200 μl of methanol (99%) for 10 min. After the removal of methanol, the individual wells were stained with 200 μl of CV (0.1%) for 25 min. The extra stain was removed, and the plates were left to dry. The amount of the CV incorporated by the adherent bacteria was resolubilized using 200 μl of 95% ethanol. Finally, OD was measured at 595 nm with a plate reader (Bio-TEK, USA). The OD values were used as an index of bacteria that adhered to the surface for establishing the biofilm. The experiment was accomplished in three replicates. The biofilm inhibition percentages were calculated as follows.
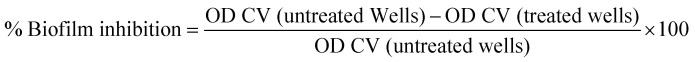


### 6.2 Biofilm disruption assay

This assay was adopted to evaluate the impact of CuNPs and AgNPs on preformed biofilms. A bacterial suspension (10 μl) was added to 96-well plates containing 190 μl MHB. To encourage bacterial growth and biofilm formation, the plates were incubated for 24 hours. Following incubation, the old media were discarded, and the individual wells were gently rinsed to remove unattached cells. The remaining biofilms were replaced with 200 μl fresh MHB containing MBC, MIC, 1/2, 1/4, and 1/8 MIC of NPs. The plates were incubated for an extra 24 hours. The following day, the media were discarded, and the wells were rinsed off to eliminate unattached bacteria and air-dried. The same method employed for biofilm inhibition was used to quantify the remaining biofilms, following CV staining. The amount of biofilm disruption in each treatment was estimated in accordance with the amount of biofilm growth in untreated wells (without NPs), defined as 100%.^39^ The percentage of biofilm disruption was calculated as follows:
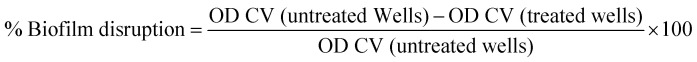


## 7 Anti-quorum-sensing assay

The assay involves the treatment of bacteria with 1/2 MIC of NPs, RNA extraction, synthesis of cDNA, and quantitative real-time PCR of quorum-sensing-associated genes, as described previously.^40^ A 96-well plate containing 190 μl MHB was inoculated with 10 μl of a bacterial suspension, treated with 1/2 MIC concentration of NPs, and then incubated at 37 °C for 24 hours. Untreated wells were incubated under the same conditions without NPs. Following incubation, the wells were vigorously pipetted, and the cells were harvested in Eppendorf tubes and centrifuged at 8000 rpm for 5 min. The AddPrep Total RNA Extraction Kit (Addbio, Korea) was used to extract the RNA from the pellet according to the protocol's instructions. A NanoDrop spectrophotometer (BIOBASE, China) was used to evaluate the concentration and purity of RNA. A one-step Addscript RT-PCR Syber mastermix (Addbio, Korea) was used, and each qRT-PCR reaction was prepared with a volume of 20 μl containing master mix (10 μl), 1 μl of forward primer (10pmol), 1 μl of reverse primer (10pmol), water up to 20 μl, and 20 ng RNA template. The reaction conditions were adjusted according to the kit's instructions as follows: reverse transcription at 50 °C for 10 min, polymerase activation at 95 °C for 3 min, 40 cycles of denaturation at 95 °C for 10 seconds and annealing/extension at 60 °C for 30 seconds. The expression of quorum-sensing genes was normalized with the expression of 16S rRNA (a reference gene). The list of primers is shown in Table 1. Gene expression levels in NP-treated and untreated conditions were calculated using the 2^−ΔΔCt^ technique as follows:ΔCt of a treated gene = Ct of a treated gene − Ct of treated 16S rRNA.ΔCt of an untreated gene = Ct of an untreated gene − Ct of untreated 16S rRNA.

**Table 1:** List of primers of quorum sensing associated genes of *P. aeruginosa*

Genes	Primers	References
LasI	Forward: 5′-CGCACATCTGGGAACTCA-3′	10
	Reverse: 5′- CGGCACGGATCATCATCT-3′	
LasR	Forward: 5′- CTGTGGATGCTCAAGGACTAC-3′	
	Reverse: 5′- AACTGGTCTTGCCGATGG-3′	
RhlI	Forward: 5′- GTAGCGGGTTTGCGGATG-3′	
	Reverse: 5′- CGGCATCAGGTCTTCATCG-3′	
RhlR	Forward: 5′- GCCAGCGTCTTGTTCGG-3′	
	Reverse: 5′- CGGTCTGCCTGAGCCATC-3′	
PqsABCDE	Forward: 5′- GACCGGCTGTATTCGATTC-3′	
	Reverse: 5′- GCTGAACCAGGGAAAGAAC-3′	
PqsR (MvfR)	Forward: 5′- CTGATCTGCCGGTAATTGG-3′	
	Reverse: 5′- ATCGACGAGGAACTGAAGA-3′	
16S (reference gene)	Forward: 5′-GAGGAAGGTGGGGATGACGT-3′	
	Reverse: 5′-AGGCCCGGGAACGTATTCAC-3′	

ΔCt of a treated gene was subtracted from the ΔCt of an untreated gene as follows:ΔΔCt = ΔCt of a treated gene − ΔCt of an untreated gene.

The relative expression of a gene was estimated as fold change using 2^−ΔΔct^.

### 7.1 Statistical analysis

The antibacterial and antibiofilm effects of AgNPs and CuNPs on bacterial strains were analyzed using GraphPad Prism version10 and the data are represented as the mean ± SD. Key statistical tests included the Brown-Forsythe, Kruskal–Wallis and one-way ANOVA, with Dunnett's post-hoc test determining significant differences. A *p* < 0.05, 0.01, and 0.001 threshold validated the results between NP-treated and untreated bacteria.

## 8 Results and discussion

### 8.1 Phenotypic identification of *P. aeruginosa*

*P. aeruginosa* was identified through conventional methods and a highly automatic BD Phoenix™ M50 identification system (Table S1). The BD Phoenix profile showed *P. aeruginosa*, and the DB Phoenix antimicrobial sensitivity revealed that the isolate was extensively drug resistant (XDR).

### 8.2 Identification of *P. aeruginosa* using 16S rRNA gene

**Fig. 1:**
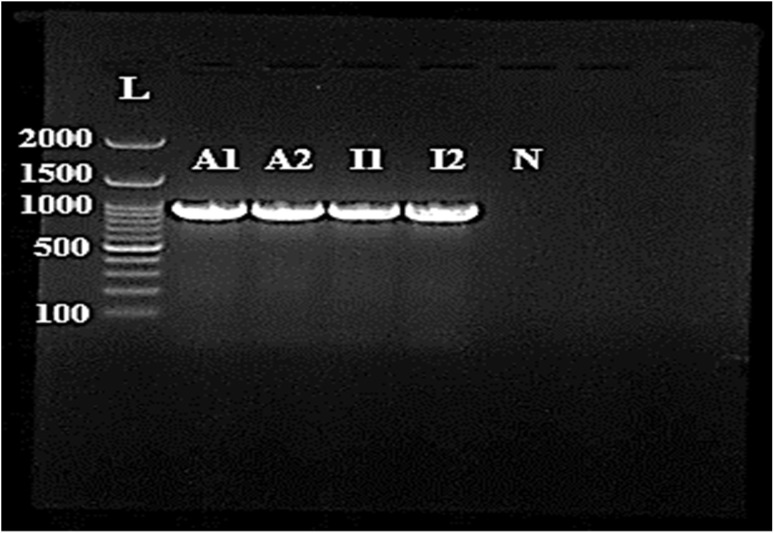
Gel electrophoresis of 950 bp PCR bands of *P. aeruginosa* was performed in 1.5% agarose and run for 50 min at 90 V. L (ladder, 100bp), A1 & A2 (*P. aeruginosa* ATCC), I1 & I2 (*P. aeruginosa* isolate), and N (negative control).

The 16S rRNA is suggested as a useful identification method and has been utilized in defining the phylogeny of species. Here, the 16S rRNA was used for the detection of the ATCC and the clinical isolate of *P. aeruginosa* using particular primers PA SS-F and PA SS-R. Both strains were recognized as *P. aeruginosa* after PCR amplification of rRNA genes with distinctive bands around 950 bp (Fig. 1). The result agreed with the previous studies.^25,26^

### 8.3 Synthesis and characterization of CuNPs and AgNPs

#### 8.3.1 Synthesis of NPs and UV-visible spectroscopy

**Fig. 2:**
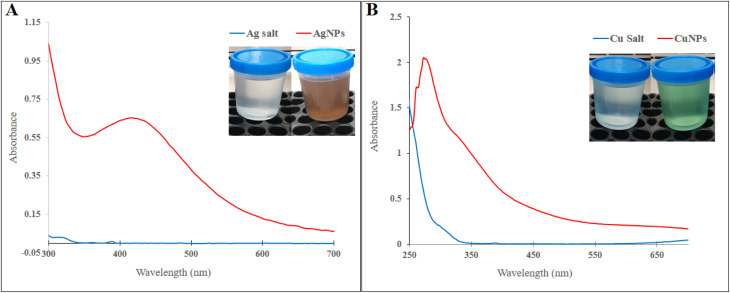
UV-visible spectra of nanoparticles. (A) AgNPs, (B) CuNPs.

*F. solani* was successfully employed in the bio-reduction of AgNO_3_ and CuNO_3_ into respective AgNPs and CuNPs after incubation of fungal biomass with the salt solutions for 6 days. The formation of AgNPs is indicated by the presence of a brown supernatant,^28^ while the formation of CuNPs is indicated by the presence of a green supernatant.^41^ There were no color changes in the negative controls. The wavelength of UV-visible spectroscopy confirmed a broad peak with maximum absorption between 415 and 420 nm, representing the surface plasmon resonance nature of the AgNPs existing in the brown supernatant (Fig. 2a). Additionally, it confirmed a maximum peak at 280 nm, representing the SPR of the CuNPs existing in the green supernatant (Fig. 2b). In this study, the absorption spectra of AgNPs were consistent with fungal-mediated AgNP synthesis.^28,42^ Previous studies clarified that the absorption peak of AgNPs is observed in the 380–450 nm range based on the particles' size, shape, and agglomeration.^43,44^ Comparable to the achieved result, the maximum absorption spectrum of CuNPs synthesized using *Trichoderma asperellum* was observed at 285 nm,^45^ and that by endophytic *Aspergillus terreus* was observed at 280 nm.^41^ Various factors such as salt concentration, particle and crystallite sizes, particle shape, and agglomeration can affect the SPR of CuNPs.^46^

### 8.4 X-ray diffraction (XRD) at different annealing temperatures and antibacterial activity of the NPs

**Fig. 3:**
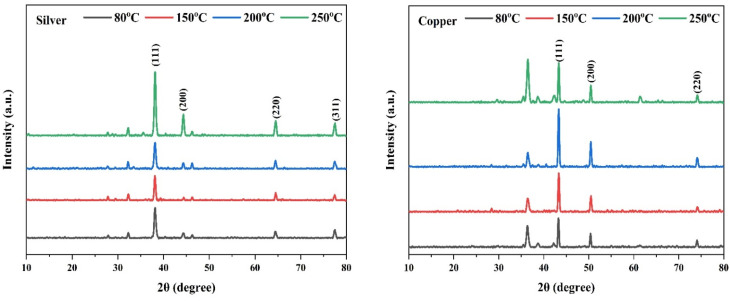
XRD patterns of AgNPs (left) and CuNPs (right) annealed at different annealing temperatures.

The diffraction patterns of AgNPs and CuNPs at different annealing temperatures (Fig. 3) revealed the crystalline structures of the NPs with a face-centered cubic (FCC) configuration. The diffraction patterns of AgNPs displayed four distinct peaks at 38.12°, 44.31°, 64.54°, and 77.53°. These peaks corresponded to 111, 200, 220, and 311 crystal planes of silver according to the JCPDS (file no, 04-0783) for silver. The diffraction patterns of AgNPs were similar to those in the previous studies.^47,48^ The diffraction patterns of CuNPs displayed three distinct peaks at 43.33°, 50.46°, and 74.15° that corresponded to (111), (200), and (220) crystal planes according to the JCPDS (file no. 80-1268) for copper. The peaks were compatible with previous research.^41,49^ Hence, they confirmed the phase purity and structural integrity of the nanoparticles. Subsequent analysis reveals that different annealing temperatures affected the crystallite size, surface area, and other physical properties of both NPs (Table S2). A previous study clarified that variation in annealing temperatures affects the physical properties of NPs, such as crystallite size, particle size and shape, intensity of diffraction peaks, and specific surface area.^50^ AgNPs annealed at 200 °C displayed the smallest crystallite size of 18.29 nm, and CuNPs annealed at 150 °C exhibited the smallest crystallite size of 26.68 nm compared to other annealing temperatures (Table S2). Additionally, AgNPs and CuNPs annealed at 200 and 150 °C respectively, demonstrated higher antibacterial activity compared to other annealing temperatures. The inhibition zone of AgNPs was bigger than that of colistin, and the inhibitory zone of CuNPs was nearly the same as that of colistin (Fig. S1 and Table 2). The possible explanation for the promising antibacterial action of both NPs may be related to the smaller crystallite and higher specific surface area. Previous studies have proven that smaller crystallites usually exhibit higher antibacterial effectiveness.^51,52^ The crystallite sizes of both NPs were smaller than those of previously mycosynthesized NPs.^32,41,53^ Owing to their smaller crystallite sizes and higher antibacterial efficiencies, AgNPs annealed at 200 °C and CuNPs annealed at 150 °C were selected for further characterization, antibacterial, antibiofilm, and anti-quorum sensing activities.

**Table 2:** The effects of different annealing temperatures on the antibacterial potential of AgNPs and CuNPs. The inhibition zone diameters were measured in mm

*Pseudomonas aeruginosa*	AgNPs (annealing temperatures) & colistin					CuNPs (annealing temperatures) & colistin				
	80 °C	150 °C	200 °C	250 °C	Colistin 10 μg	80 °C	150 °C	200 °C	250 °C	Colistin 10 μg
ATCC 9027	11	10	19	11	15	10	14	7	6	15
Clinical isolate	14	11	17	11	13	0	9	0	0	14

### 8.5 SEM and TEM

**Fig. 4:**
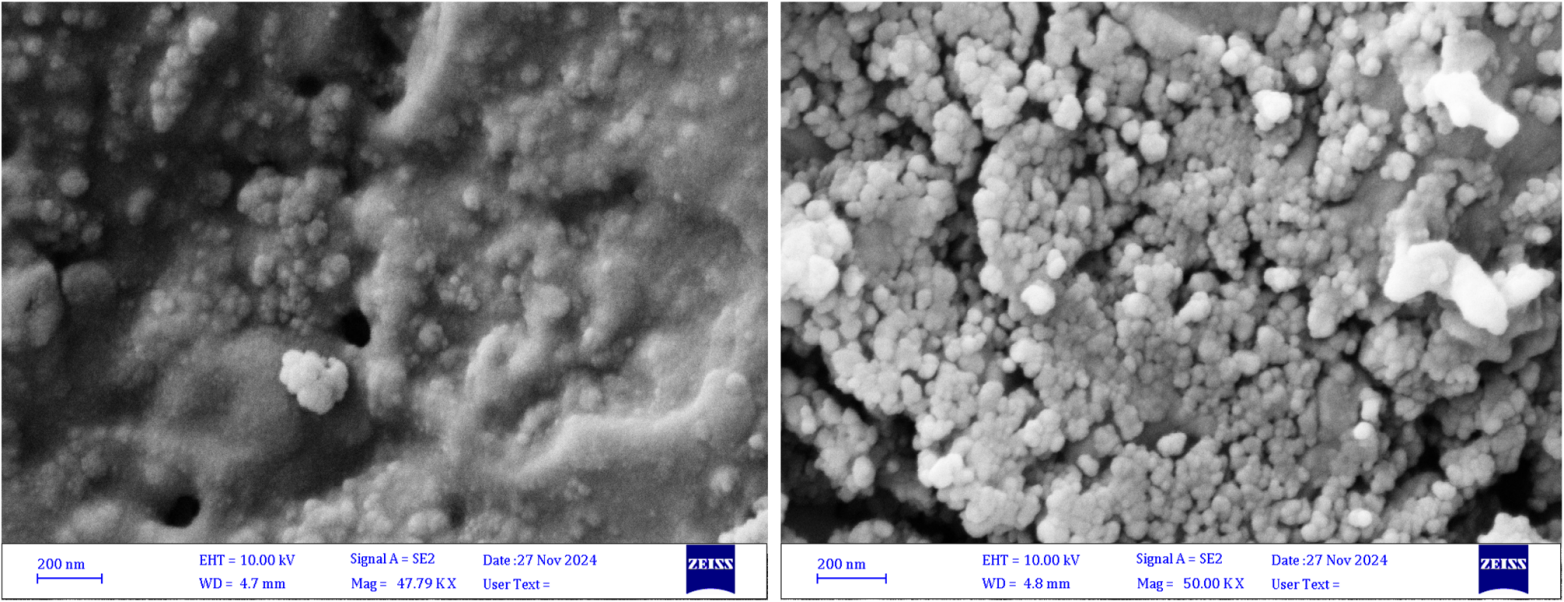
SEM images showed biogenic spherical-shaped AgNPs (left) and CuNPs (right) synthesized using *F. solani*.

**Fig. 5:**
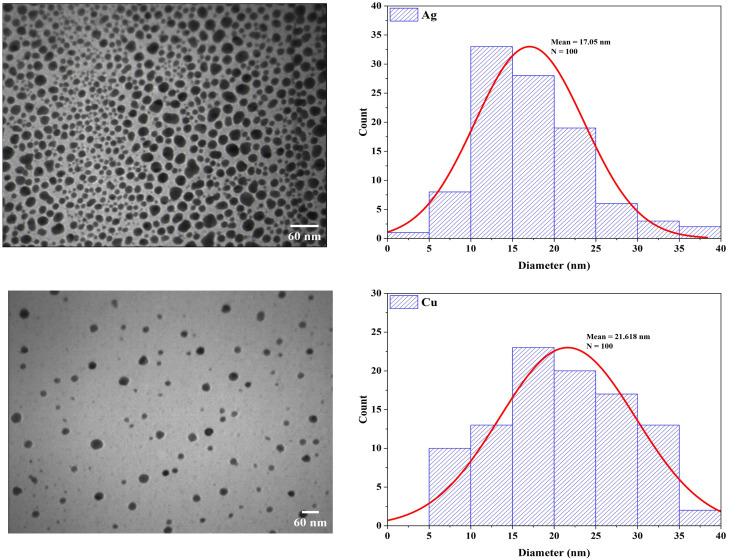
TEM images of biogenic AgNPs and CuNPs showed spherical-shaped and monodispersed particles, along with a histogram of particle size distribution.

SEM images of AgNPs and CuNPs displayed spherical NPs with the presence of agglomerates as small clusters, especially in the powders of AgNPs, and fewer agglomerates in CuNPs. The clusters made the particles larger in the SEM images (Fig. 4). Previous studies supported the formation of CuNPs and AgNPs with the evidence of agglomerates.^53,54^ TEM confirmed the formation of spherical and nearly spherical NPs in various sizes with smooth surfaces and uniform distribution (monodisperse) without agglomeration. The histogram of particle size distribution revealed that the average diameter of 100 particles of AgNPs was 17 nm and mostly between 10 and 15 nm, while the average diameter of 100 particles of CuNPs was 21 nm and mostly between 15 and 20 nm (Fig. 5). The spherical shapes and size distribution of NPs were relatively comparable with other studies.^54,55^

### 8.6 EDS

**Fig. 6:**
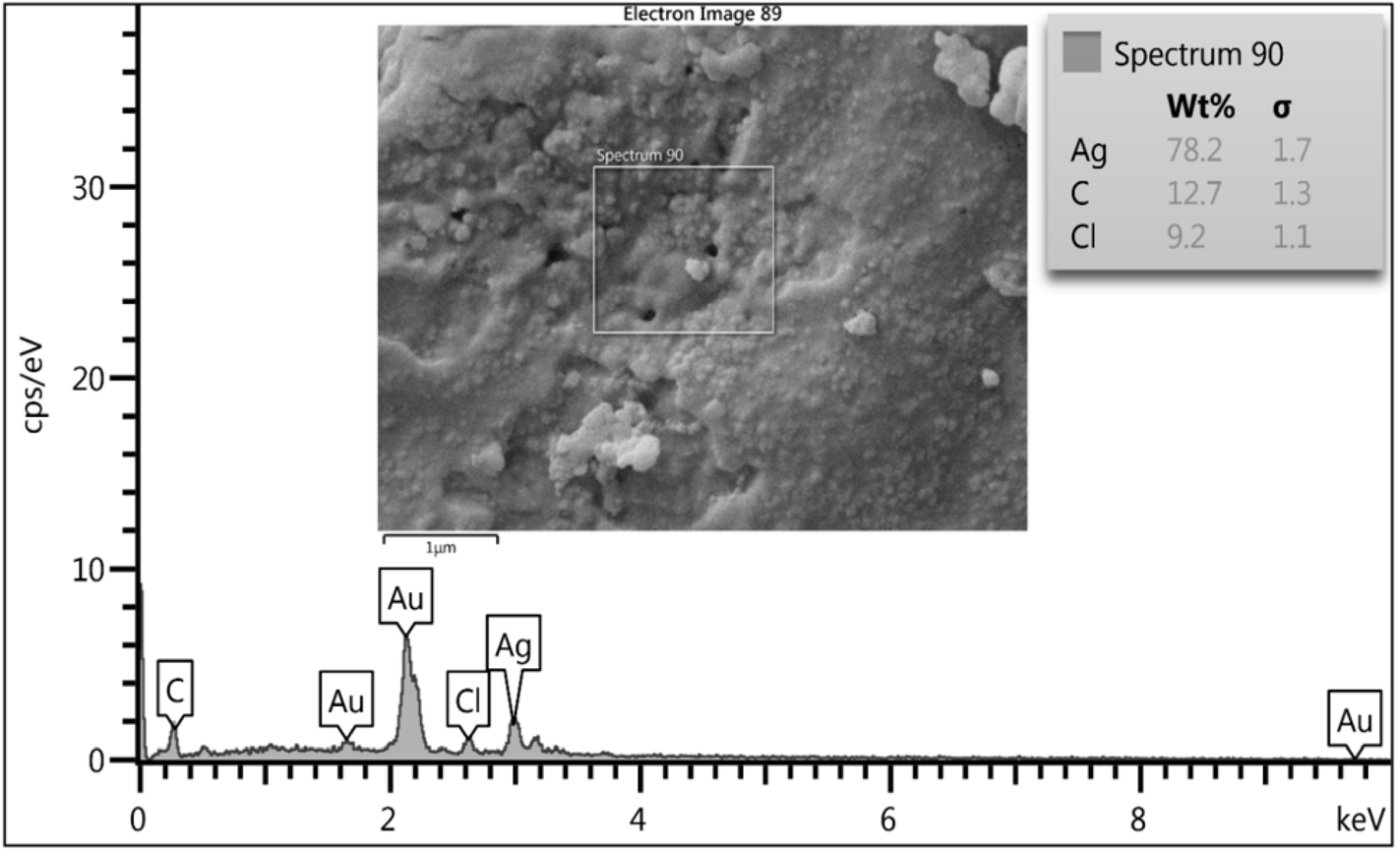
The elemental composition of biogenic AgNPs synthesized using *F. solani*.

**Fig. 7:**
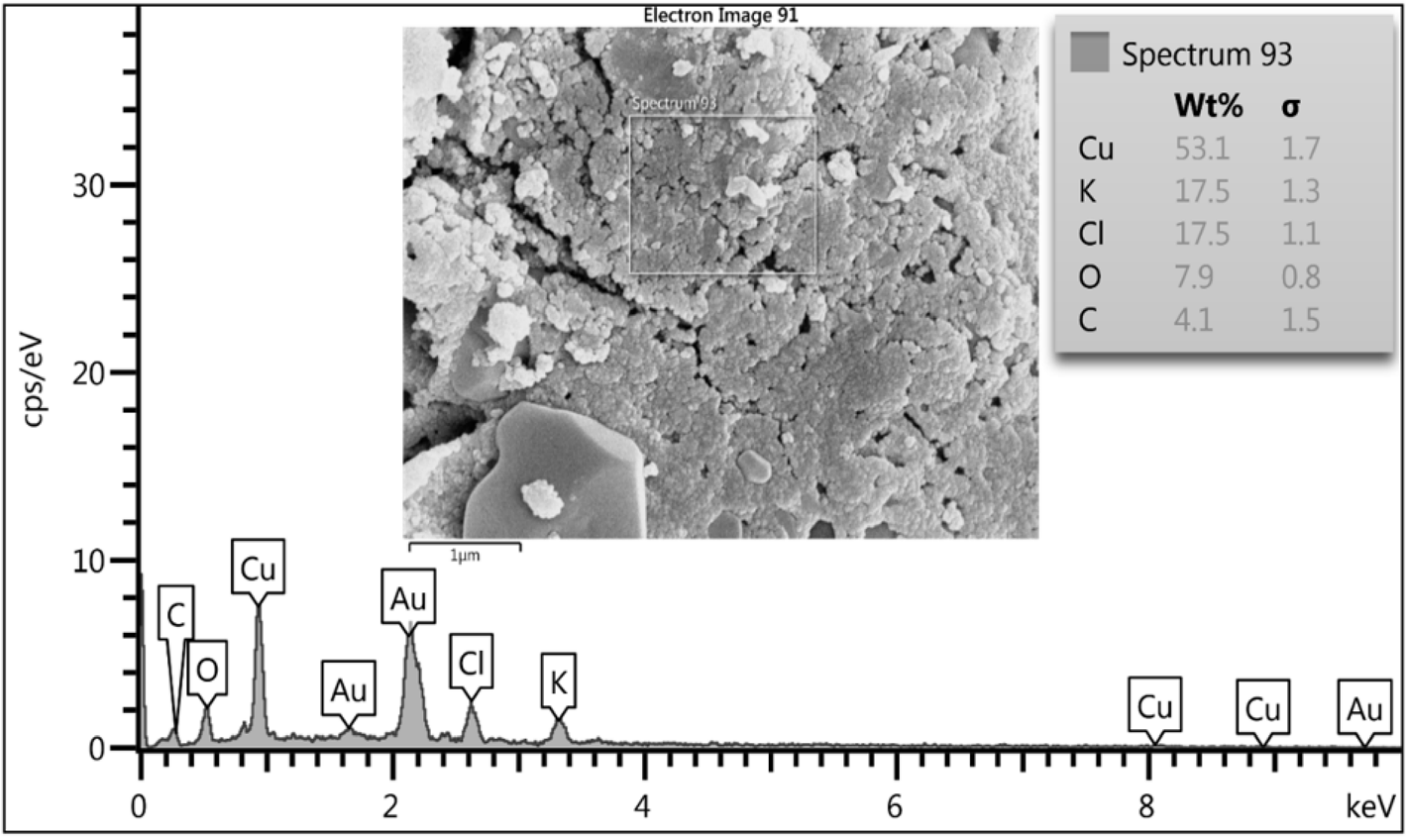
The elemental composition of biogenic CuNPs synthesized using *F. solani*.

Elemental compositions of biogenic NPs were analyzed using EDS, revealing that silver and copper are the major constituents in the nanostructures. The peak observed at 3 keV suggests the fabrication of metallic AgNPs.^47^ In addition to silver, weaker peaks at 0.3 and 2.6 keV were observed, corresponding to carbon (C) and chlorine (Cl), respectively. The total weight percentage of silver was 78.2%, whereas that of C was 12.7% and Cl was 9.2% (Fig. 6). The EDS of the biogenic CuNPs displayed copper metal absorption peaks at 1, 8, and 9 keV, while that of oxygen (O) was near 0.5 keV, and they were similar to the EDS signals of previously biosynthesized CuNPs.^41,56^ Other absorption peaks, such as potassium (K), carbon (C), and chlorine (Cl), were observed at 3.3, 0.3, and 2.6 keV, respectively. The weight percentages for Cu, O, C, Cl, and K were 53.1%, 7.9%, 4.1%, 17.5%, and 17.5%, respectively (Fig. 7). The presence of C, Cl, and K peaks in the EDS corresponded to the components of fungal biomass.^57^ The Au peaks corresponded to the gold element used in the EDS analysis.

### 8.7 FTIR spectroscopy

**Fig. 8:**
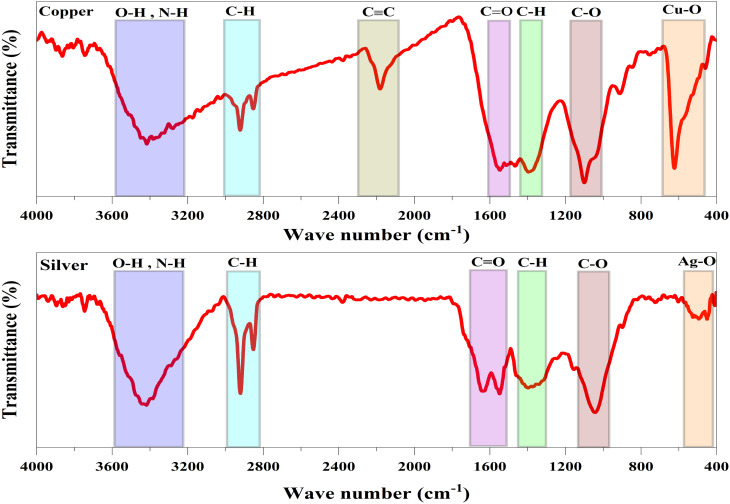
FTIR spectra of biogenic CuNPs and AgNPs displaying the presence of various functional groups.

The fungal biomass's potential functional groups that contributed to the synthesis, capping, and stabilization of the NPs were analyzed using FTIR (Fig. 8). FTIR spectra of the biogenic CuNPs and AgNPs displayed remarkable absorption peaks as follows: Cu peak (O–H or N–H => Stretching => 3419) and Ag peak (O–H or N–H => Stretching => 3428) corresponded to O–H stretching of phenols and alcohols or N–H stretching mode in amines found in amino acids of proteins.^41,53,58^ Cu peak (C–H => Stretching => 2833–2980) and Ag peak (C–H => Stretching => 2828–2981) indicated the vibrational stretching of C–H found in aliphatic hydrocarbons (alkanes).^59,60^ Cu peak (C≡C => Stretching => 2180) corresponds to the alkynes.^61^ Cu peak (C=O => Stretching => 1545) and Ag peak (C=O => Stretching => 1534–1681) corresponded to C=O vibrational stretching of polysaccharides^62^ or vibrational stretching in the amide bond of the fungal proteins.^63^ Cu peak (C–H => Bending => 1392) and Ag peak (C–H => Bending => 1383) corresponded to C–H bending of aldehyde groups.^48,64^ Cu peak (C–O => Stretching => 1098) and Ag peak (C–O => Stretching => 1042) represented C–O stretching vibration in primary alcohols.^48,65^. Cu–O => Stretching => 621.50 and Ag–O => Stretching => 471 indicated the formation of CuNPs,^41,66^ and AgNPs,^53^ respectively. Various functional groups comprising phenols, alcohols, amines, amides, polysaccharides, alkanes, alkynes, and aldehydes confirmed the successful formation and stabilization of NPs through *F. solani*.

### 8.8 Antibacterial activity

**Fig. 9:**
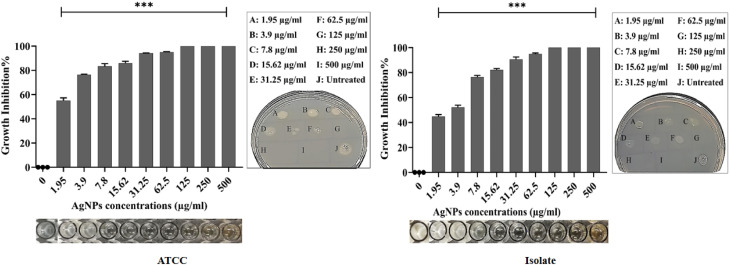
Dose-dependent growth inhibition% of AgNPs against the ATCC and the isolate of *P. aeruginosa*. The spot assay was performed on both treated and untreated wells using nutrient agar. Data are given as the mean ± SD of triplicates, and the difference between treated and untreated bacteria was statistically significant (***p < 0.001).

**Fig. 10:**
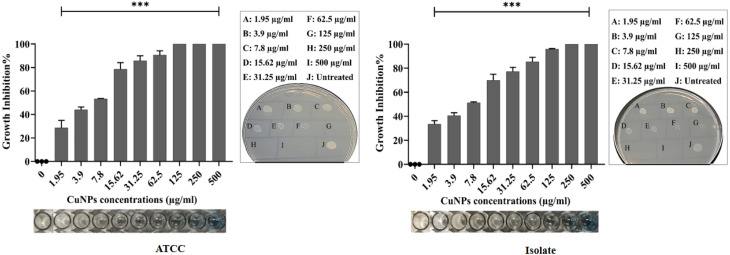
Dose-dependent growth inhibition% of CuNPs against the ATCC and the isolate of *P. aeruginosa*. The spot assay was performed on both treated and untreated wells using nutrient agar. Data are given as the mean ± SD of triplicates, and the difference between treated and untreated bacteria was statistically significant (***p < 0.001).

The biogenic NPs revealed promising antibacterial action against *P. aeruginosa* ATCC and XDR strains. The growth inhibition percentages were calculated based on the concentration of NPs (Fig. 9 and 10). The growth inhibition of bacteria treated with NPs differs significantly from that of untreated bacteria (***p < 0.001). The MIC and MBC of AgNPs against the ATCC strain were 31.25 and 125 μg mL^−1^, respectively, whereas against the isolate they were 62.5 and 125 μg mL^−1^. The MIC and MBC of CuNPs against the ATCC strain were 62.5 and 125 μg mL^−1^, respectively, whereas against the isolate they were 125 and 250 μg mL^−1^. Compatible with the present results, the MIC and MBC of AgNPs ranged between 50 and 250 μg mL^−1^ in previous studies.^67–70^ Here, the MIC and MBC of AgNPs were less than those in a previous study.^71^ Inversely, the values were higher than in ref. 72. The MIC and MBC of biogenic CuNPs were lower than those in a previous study^57^ and comparable with those in ref. 73. These variations in the antimicrobial activities of NPs in the present study and previous studies are attributed mainly to the variation in the size of nanoparticles. It has been proven that particles with smaller sizes provide more potent antibacterial action due to higher surface area and faster ion release.^74^ Other related factors include synthetic methods, shapes, and charges of the NPs, agglomeration, nature of capping agents, bacterial strains, and their response.^70,75^ In the present study, both NPs had bactericidal action against the target bacteria. Owing to a greater surface area, strong reactivity, and the attraction of negative charges of bacterial cell wall and membrane components, AgNPs can penetrate the cell more effectively than bulky substances, leading to cell rupture and/or disruption of essential cellular functions such as permeability and respiration. Similarly, membrane destabilization by AgNPs causes intracellular ATP depletion and cell death.^71^ AgNPs can also damage bacterial cells by interacting with compounds containing sulfur and phosphorus, particularly protein and DNA. Additionally, AgNPs release Ag+ ions into the cytoplasm, which has bactericidal action.^76,77^ Further mechanisms involve pore formation and the ROS generation, such as superoxide ions, hydroxyl ions, and hydrogen peroxide, which oxidize biomolecules.^78^ CuNPs produce highly toxic ions after breakdown inside the bacterial cells. The liberated ions react with the thiol group, thereby stopping protein function.^41^ Likewise, ion accumulation interferes with the proper function of the membrane permeability.^79^ Moreover, CuNPs release a large quantity of ROS, causing lipid peroxidation, DNA, and protein degradation.^80^ The morphological change and pore formation in the bacterial cell wall were also observed when treated with CuNPs.^81^

### 8.9 The biofilm formation ability of *P. aeruginosa* strains

**Fig. 11:**
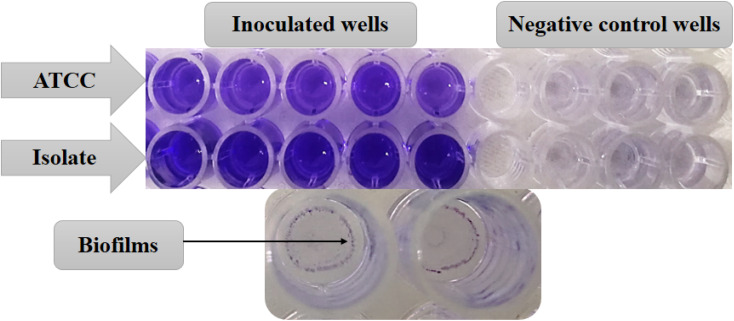
Biofilm formation by strains of *P. aeruginosa* using crystal violet.

The ability of the ATCC and the clinical isolate of *P. aeruginosa* to produce biofilm was assessed through the estimation of OD of CV in inoculated and negative control wells (Fig. 11). The obtained results were classified semi-quantitatively into non-biofilm producer, weak, moderate, and strong according to the following calculations:

(1) Biofilm formation = OD of CV (inoculated wells) − OD of CV (negative control wells). Mean OD values of inoculated wells for ATCC and isolate were 1.7 & 2, respectively, while mean OD values of negative controls were 0.15 & 0.16, respectively. The OD values of both strains were more than 10 times greater than the OD of the negative controls.

(2) OD value of a tested strain = average OD of a strain − cutoff value of negative control (ODc). ODc = average OD negative control + (3 × SD of negative control). The average OD of ATCC was 1.7, and its ODc was 0.21. The average OD of the isolate was 2, and its ODc was 0.23. OD value of ATCC strain = 1.49 > 4*x*ODc. OD value of isolated strain = 1.77 > 4*x*ODc. Based on the above calculations, both strains were strong biofilm producers.^36,37^

### 8.10 Biofilm inhibition and disruption

**Fig. 12:**
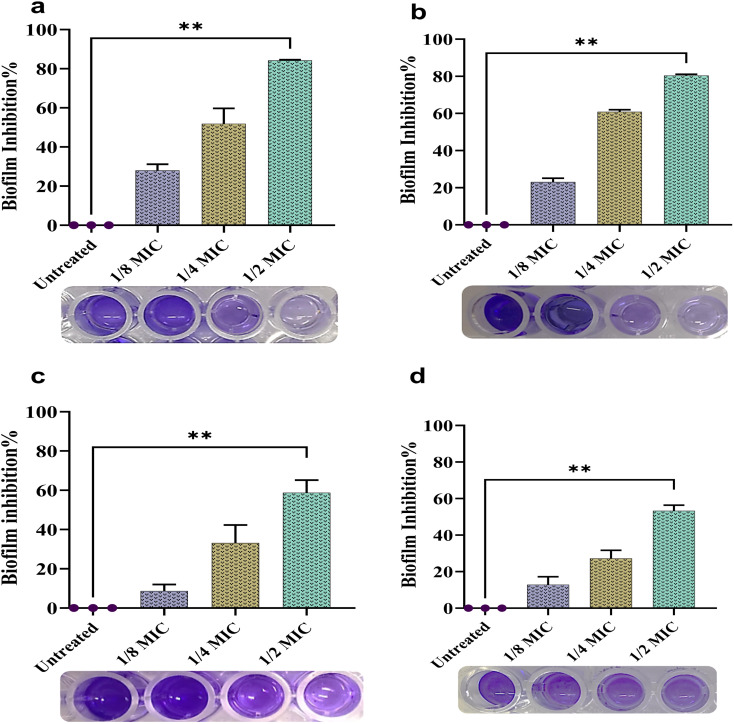
Biofilm inhibition% of NPs at 1/8, 1/4 & 1/2 MIC concentrations against *P. aeruginosa*: (a) ATCC-AgNP treated, (b) isolate-AgNP treated, (c) ATCC-CuNP treated, (d) isolate-CuNP treated. The data are given as means ± SD. The significant differences are represented as **p < 0.01.

**Fig. 13:**
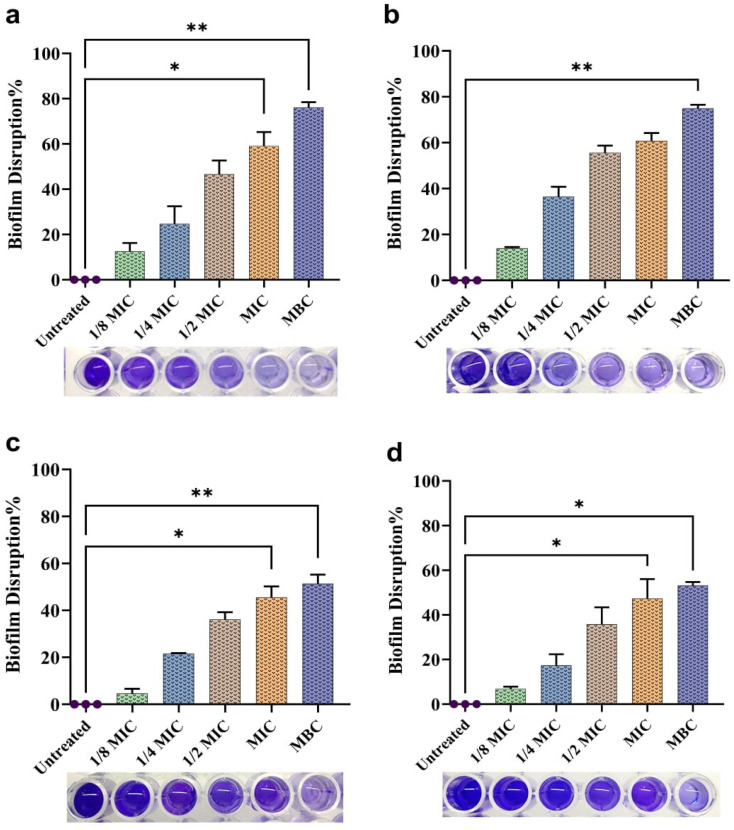
Biofilm disruption% of NPs at 1/8, 1/4 & 1/2 MIC, MIC & MBC concentrations against *P. aeruginosa*: (a) ATCC-AgNP treated, (b) isolate-AgNP treated, (c) ATCC-CuNP treated, (d) isolate-CuNP treated. The data are given as means ± SD. The significant differences are represented as * & ** = *p* < 0.05 & <0.01.

Nearly 65–80% of infections were caused by biofilm-producing bacteria, among them, *P. aeruginosa* is the most common.^82^ Here, biogenic NPs demonstrated significant biofilm inhibition potential against *P. aeruginosa* at sub-MIC concentrations compared to the control (**p < 0.01), Fig. 12. The percentages of biofilm inhibition of AgNPs at 1/8MIC, 1/4MIC, and 1/2MIC against the ATCC were 27.97 ± 2.51, 51.87 ± 3.79, and 84.23 ± 0.44, respectively. In contrast, the inhibition percentages of the same concentrations against the isolate were 23.03 ± 1.63, 60.8 ± 1.6, and 80.43 ± 0.9. The percentages of biofilm inhibition of CuNPs at 1/8MIC, 1/4MIC, and 1/2MIC against the ATCC were 8.67 ± 1.4, 32.13 ± 3.2, and 58.77 ± 3.2, respectively, while against the isolate they were 8.00 ± 1.4, 30.57 ± 1.3, and 53.3 ± 2.07. Indeed, a few NPs are known to be effective in disrupting preformed biofilms. Also, a restricted number of antibiotics can disrupt preformed biofilms. Here, the biogenic NPs significantly disrupted preformed biofilms at sub-MIC, MIC, and MBC concentrations compared to the control (*p & **p < 0.05 & 0.01), Fig. 13. The percentage of biofilm disruption of AgNPs at 1/8MIC, 1/4MIC, 1/2MIC, MIC, and MBC against ATCC were 12.57 ± 1, 24.70 ± 1.4, 46.6 ± 1.9, 59.07 ± 2.1, and 76.13 ± 0.7, respectively, whereas against the isolate they were 14 ± 0.8, 36.47 ± 1.4, 55.53 ± 0.5, 60.77 ± 1.04, and 74.97 ± 1.5. The percentages of biofilm disruption of CuNPs at 1/8MIC, 1/4MIC, 1/2MIC, MIC, and MBC against the ATCC were 4.67 ± 0.6, 21.53 ± 0.5, 36.10 ± 1.8, 45.57 ± 1.9, and 51.40 ± 1.11 respectively, whereas against the isolate they were 6.83 ± 1.7, 17.33 ± 1.5, 35.83 ± 1.8, 47.33 ± 2.5, and 52.17 ± 0.7. Here, the biofilm inhibition of AgNPs at sub-MIC concentrations was relatively higher than in the previous studies.^30,75^ A study^12^ showed that 1/2 MIC of AgNPs inhibited biofilms in several isolates of *P. aeruginosa* by 2–89%. In another study, there is no substantial change in biofilm inhibition under sub-MIC treatment.^83^ Inversely, there is an increase in biofilm formation at sub-MIC treatment.^69^ In a previous study, the biofilm disruption activity of AgNPs at 1/4 MIC to 16 MIC against ATCC and clinical isolates such as *P. aeruginosa* ranged from 10 to 85%.^30^ In another study, different-sized AgNPs, 8 and 35 nm, distracted the preformed biofilm of *P. aeruginosa* by 90% and 52% at 600 μg mL^−1^.^84^ Here, the biofilm inhibition of CuNPs at 1/2 MIC was more than 50%. In a previous study, the biofilm inhibition of CuNPs was 45% at 1/2 MIC.^85^ In another study, CuNPs synthesized by *P. chrysogenum* did not affect the formation of biofilm by *P. aeruginosa* even at a milligram dose.^57^ In the present work, the effective biofilm inhibition and disruption of both NPs may be due to the smaller particle and crystallite sizes having a greater surface area to interact and adhere to bacterial cells, in turn preventing the bacterial attachment to surfaces, inhibiting the initial phase of biofilm development.^86^ Furthermore, smaller NPs with a higher surface area can easily interact with biofilm constituents, thereby having a better chance of penetration and dispersion throughout the biofilm.^87^ Also, NPs cause disruption of membrane integrity, alteration of cell morphology, and rearrangement in the biofilm.^88^ Additionally, NPs neutralize the extracellular polymeric substance (EPS) and adhere to the cell membrane, thereby inactivating enzymes involved in EPS biosynthesis.^16^ The generation of ROS by NPs is another possible mechanism to inhibit the biofilm.^3^

### 8.11 The effect of NPs on quorum sensing (QS) associated genes

**Fig. 14:**
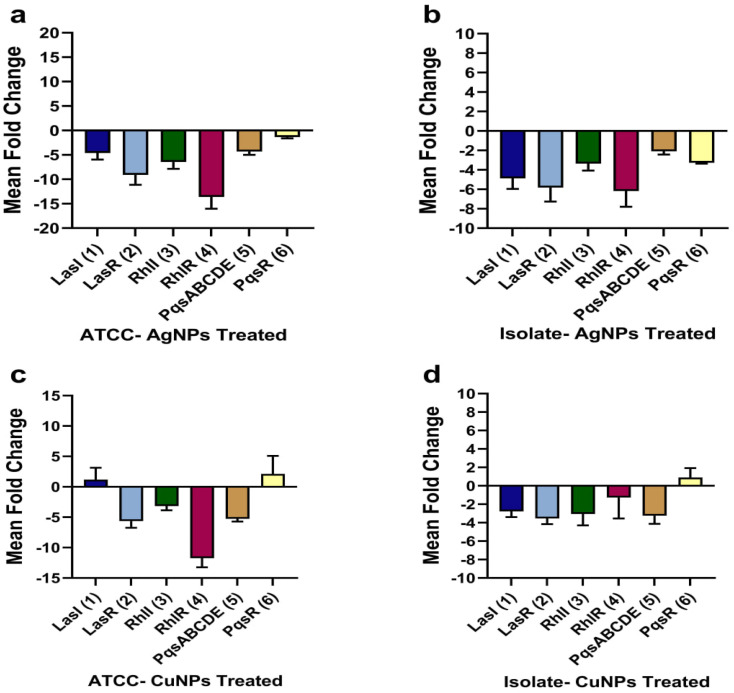
Fold change downregulation (−) or upregulation (+) in the quorum sensing associated genes of *P. aeruginosa* after treatment with 1/2 MIC of AgNPs and CuNPs, the average fold change (*n* = 3) and standard deviation.

The calculation of the threshold cycle (Ct) was used to detect the fold change in the expression of genes, including LasI/LasR, RhlI/RhlR, and PqsABCDE/PqsR. Initially, the expression of genes in 1/2 MIC-treated and untreated bacteria was normalized to the 16S rRNA as a reference gene and then analyzed as fold changes using the 2^−ΔΔCt^ technique (Fig. 14). AgNPs downregulated the LasI, LasR, RhlI, RhlR, PqsABCDE, and PqsR genes of the ATCC by 4.63, 9, 6.46, 13, 4.32, and 1.4 fold, respectively, and downregulated the genes in the isolate by 4.88, 6, 3.36, 6.18, 2.09, and 3.27 fold. In the recorded results, the downregulation of QS genes was relatively higher than that of ref. 89 and ref. 12, where the plant-based and chemical-based synthesis of AgNPs downregulated the quorum-sensing genes of *P. aeruginosa* at 1/2 MIC. In another study, the AgNPs from *R. arrhizus* suppressed the QS regulatory genes.^90^ In contrast to the present study, *F. oxysporum*-mediated synthesis of AgNPs increased the expression of QS genes at sub-MIC treatment.^83^ CuNPs downregulated the ATCC LasR, RhlI, RhlR, and PqsABCDE genes by 5.63, 3.17, 11, and 5.25 fold, respectively, whereas the LasI and the PqsR were upregulated by 1 and 2 fold, respectively. CuNPs downregulated LasI, LasR, RhlI, RhlR, and PqsABCDE genes of the isolate by 2.78, 3.54, 3, 1.3, and 3.24 fold, whereas, the PqsR was upregulated by less than a fold. Indeed, there is little work on the anti-quorum-sensing effect of CuNPs against *P. aeruginosa*. The present study highlights interesting findings concerning the potential activity of CuNPs not only as antibacterial and antibiofilm agents but also as an anti-quorum-sensing inhibitory agent. Here, the promising anti-quorum-sensing activity of both NPs may be related to several factors such as smaller particle and crystallite sizes, spherical shapes, and higher surface area, which facilitate the internalization of the particles into cells and consequently interrupt QS gene expression by inhibiting cytoplasmic components and protein functions.^91^ Furthermore, small particle and crystallite sizes provide sufficient surface area to bind with signaling molecules in QS, leading to inhibition of QS and associated virulence factors.^69^ Additionally, NPs could release ions after being transported into bacterial cells, impeding proteins and enzymes required for the development of QS.^92^ Eventually, the generation of ROS by NPs could disrupt QS gene expression.^3^

## 9 Conclusion

In the present study, biogenic AgNPs and CuNPs were successfully achieved through a sustainable, biocompatible, inexpensive, and eco-friendly approach, utilizing *Fusarium solani* as a bioreducing factory. The XRD confirmed the formation of 18–26 nm crystallite NPs with a cubic-centered configuration. The synthesis of spherical NPs with average particle sizes of 17 and 21 nm was confirmed by TEM. In the EDS profile, Ag and Cu represented the major constituents in the AgNPs and CuNPs. The FTIR verified the contribution of different functional groups in the bioreduction and stabilization of NPs. The promising antibacterial potential of biogenic CuNPs and AgNPs against *Pseudomonas aeruginosa* suggests their potential application as alternative antibacterial agents for combating drug-resistant pathogens where conventional treatments have failed. To the best of our knowledge, this is the first report demonstrating the dual activity of *F. solani*-mediated AgNPs and CuNPs in simultaneously inhibiting biofilm formation and quorum sensing in an XDR strain of *P. aeruginosa*, clarifying the possibility of being manipulated in the food industry, cosmetics, and dental care.

## Author contributions

Conceptualization and original draft, K. A. K. and S. M. M.; manuscript preparation, methodology, investigation, and formal analysis, K. A. K.; reviewing, editing, validation and supervision, S. M. M.

## Conflicts of interest

The authors declare no competing interests.

## Supplementary Material

NA-OLF-D5NA00898K-s001

## Data Availability

All data generated and analyzed throughout this study are contained within this manuscript and its supplementary information (SI). No external datasets have been deposited, as all relevant data supporting the findings are available inside the submission. Supplementary information is available. See DOI: https://doi.org/10.1039/d5na00898k.
