# Supplementary material for: Eco-friendly nanoparticles from Fusarium solani suppress biofilms and quorum sensing in Pseudomonas aeruginosa
Source: Nanoscale Adv. 2025 Nov 10;8(1):80–96. doi: 10.1039/d5na00898k (PMC12598413; doi:10.1039/d5na00898k)
Supplement: NA-008-D5NA00898K-s001 [file NA-008-D5NA00898K-s001.pdf]

Supplementary Information (SI) for Nanoscale Advances.
This journal is © The Royal Society of Chemistry 2025

Eco-friendly Nanoparticles from *Fusarium solani* Suppress Biofilms and Quorum Sensing in *Pseudomonas aeruginosa*

Karokh Ali Khdir [1], Sirwan Muhsin Muhammed[2]

[1]Biology Department, College of Education, University of Sulaimani, Sulaymaniyah, Kurdistan Region, 46001, Iraq

[2]Biology Department, College of Science, University of Sulaimani, Sulaymaniyah, Kurdistan Region, 46001, Iraq

Corresponding author

Karokh Ali Khdir

[1]karokh.khdir@univsul.edu.iq

[2]sirwan.muhammed@univsul.edu.iq

**Identification tests**	**Results**
Gram staining	Gram negative bacilli, singly or in pairs
Cetrimide agar	The colonies were round, convex and mucoid with blue-green and yellow-green pigmentations
MacConkey agar	Colonies were flat, smooth and non- lactose fermenter with a little pigmentation
Catalase	Positive
Oxidase	Positive
Highly automatic BD Phoenix™ M50 identification system	Showed *P. aeruginosa*

Table S1. List of phenotypic characteristics used in identification of *P.aeruginosa*

**Samples**	**Annealing (°C)**	**D (nm)**	**δ (nm^{-2})**	**ε**	**d-spacing (Å)**	**ρ_x (g/cm^3)**	**SSA (m^2/g)**	**Porosity (%)**
**AgNPs**	80	18.49	0.00293	0.00574	2.356	10.53	30.82	97.15
	150	22.34	0.00200	0.00475	2.358	10.51	25.56	97.03
	**200**	**18.29**	**0.00299**	**0.00580**	**2.358**	**10.52**	**31.19**	**97.05**
	250	20.07	0.00248	0.00528	2.356	10.53	28.40	97.04
**CuNPs**	80	29.23	0.00117	0.00322	2.089	8.91	23.03	96.19
	**150**	**26.68**	**0.00141**	**0.00352**	**2.085**	**8.96**	**25.11**	**96.65**
	200	27.18	0.00135	0.00345	2.086	8.95	24.67	96.42

	250	28.33	0.00125	0.00331	2.086	8.95	23.65	96.31

Table S2. Crystallite size (D), dislocation (δ), strain (ε), d-spacing (Å), X-ray density (ρ_x), specific surface area (SSA), and porosity percentage of AgNPs and CuNPs annealed at different annealing temperatures.

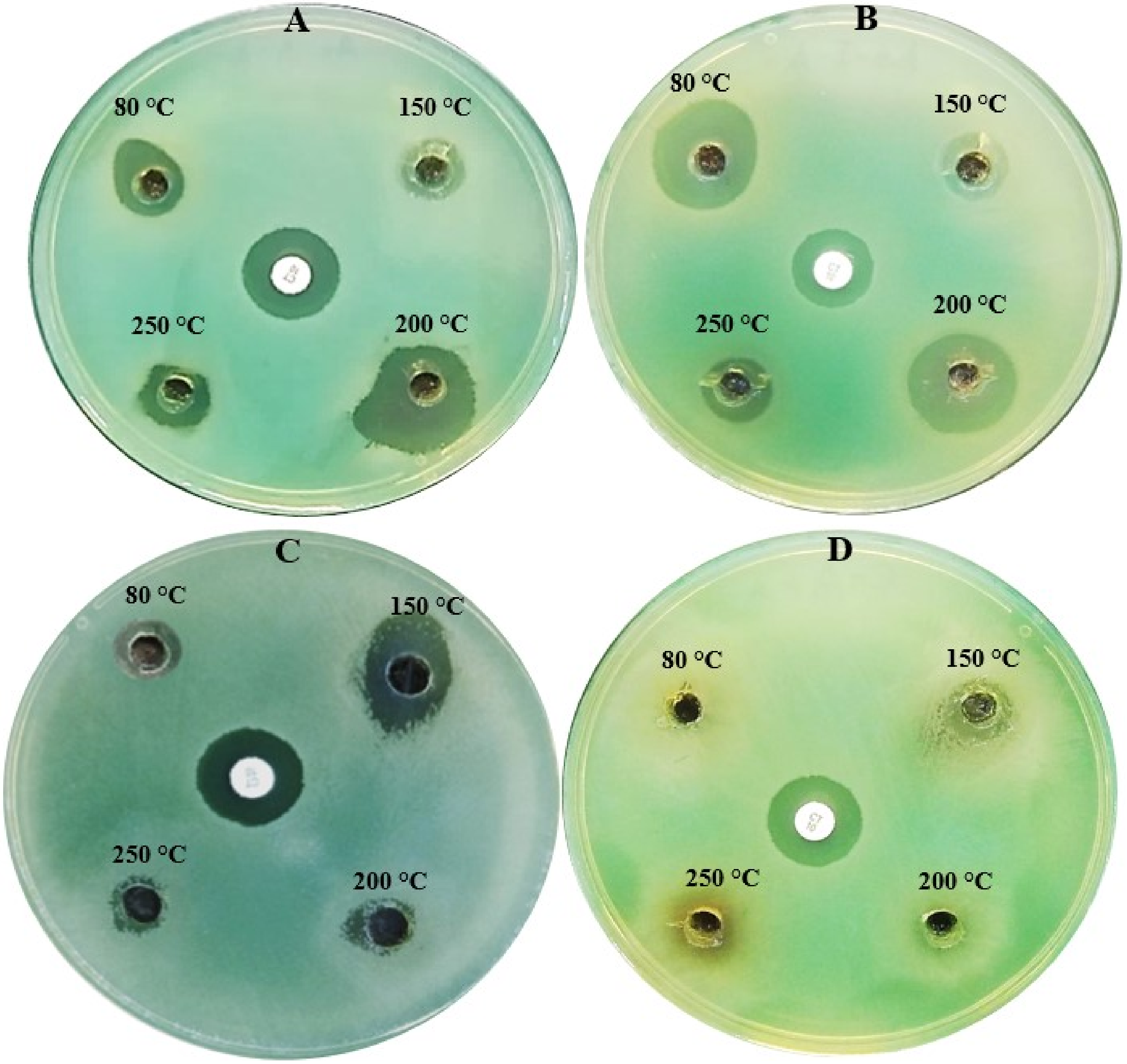

Figure S1. Antibacterial activity of AgNPs and CuNPs at different annealing temperatures of 80, 150, 200, and 250°C using well diffusion agar method. A (AgNPs against ATCC), B (AgNPs against isolate), C (CuNPs against ATCC), and D (CuNPs against isolate). Positive control is colistin (10 µg/disc).
